# Knowledge, Attitudes, Perception and Reported Practices of Healthcare Providers on Antibiotic Use and Resistance in Pregnancy, Childbirth and Children under Two in Lao PDR: A Mixed Methods Study

**DOI:** 10.3390/antibiotics10121462

**Published:** 2021-11-27

**Authors:** Vanphanom Sychareun, Amphoy Sihavong, Anna Machowska, Xanded Onthongdee, Kongmany Chaleunvong, Bounxou Keohavong, Jaran Eriksen, Claudia Hanson, Manivanh Vongsouvath, Gaetano Marrone, Annelie Brauner, Mayfong Mayxay, Sengchanh Kounnavong, Cecilia Stålsby Lundborg

**Affiliations:** 1 Faculty of Public Health, University of Health Sciences (UHS), Vientiane 7444, Laos; vsychareun@gmail.com; 2 Vientiane Capital Health Department, Ministry of Health, Vientiane 01030, Laos; amphoys@hotmail.com; 3 Department of Global Public Health, Karolinska Institutet, 17177 Stockholm, Sweden; jaran.eriksen@ki.se (J.E.); claudia.hanson@ki.se (C.H.); gaetano.marrone@ki.se (G.M.); cecilia.stalsby.lundborg@ki.se (C.S.L.); 4 Lao Tropical and Public Health Institute, Ministry of Health, Vientiane 01030, Laos; xanded.zaii@gmail.com (X.O.); sengchanhkounnavong@hotmail.com (S.K.); 5 Institute of Research and Education Development, UHS, Ministry of Health, Vientiane 01030, Laos; kchaluenvong@gmail.com (K.C.); mayfong@tropmedres.ac (M.M.); 6 Food and Drug Department, Ministry of Health, Vientiane 01030, Laos; kbounxou@yahoo.com; 7 Department of Infectious Diseases/Venhalsan, Stockholm South General Hospital, 11883 Stockholm, Sweden; 8 Lao-Oxford-Mahosot Hospital-Welcome Trust Research Unit (LOMWRU), Microbiology Laboratory, Mahosot Hospital, Vientiane 01000, Laos; Manivanh@tropmedres.ac; 9 Department of Microbiology, Tumor and Cell Biology, Karolinska Institutet, 17177 Stockholm, Sweden; annelie.brauner@ki.se; 10 Division of Clinical Microbiology, Karolinska University Hospital, 17164 Solna, Sweden; 11 Centre for Tropical Medicine and Global Health, University of Oxford, Oxford OX3 7LG, UK

**Keywords:** antibiotic, antibiotic resistance, healthcare provider, knowledge, attitude, practice, pregnancy, delivery, children under two, Lao PDR

## Abstract

Background: Overuse and misuse of antibiotics contribute unnecessarily to antibiotic resistance (ABR), and are thereby global health threats. Inappropriate prescriptions of antibiotics during pregnancy, delivery and early childhood are widespread across the world. This study aimed to assess knowledge, attitudes, and reported practices of healthcare providers (HCPs) and to explore their perceptions regarding antibiotic use and ABR related to pregnancy, childbirth, and children under two in Lao PDR. Methods: This is a mixed methods study with data collection in 2019 via structured interviews among 217 HCPs (medical doctors/assistant doctors, midwives/nurses, pharmacists/assistant pharmacists and drug sellers), who prescribed/dispensed antibiotics in one rural and one urban district in Vientiane province and individual qualitative interviews with 30 HCPs and stakeholders. Results: Of the HCPs, 36% had below average knowledge regarding antibiotic use and ABR, and 67% reported prescribing antibiotics for uncomplicated vaginal delivery. Half of the HCPs did not believe that their prescribing contributed to ABR, and only 9% had participated in antibiotic education. Conclusion: A substantial number of HCPs had suboptimal knowledge and prescribed antibiotics unnecessarily, thereby contributing to ABR. Continuous education and regular supervision of HCPs is recommended to improve the use of antibiotics related to pregnancy, childbirth, and young children.

## 1. Introduction

Antibiotics are lifesaving interventions allowing management of infections during pregnancy, delivery and the postpartum period, leading to reduced maternal and infant mortality [1]. However, unnecessary or irrational use of antibiotics contributes to the development and spread of antibiotic resistance (ABR), which is currently considered as one of the most pressing global public health threats [2,3]. The rational use of medicines occurs when a correctly selected medicine is prescribed for appropriate indications, in doses that meet their own individual requirements, for an adequate period of time, at the lowest cost for both the patients and the society [4]. Several studies have shown that inappropriate antibiotic use is widespread across the globe [5,6,7,8,9,10,11,12]. 

Maternal infections in pregnancy are common and several pre-existing conditions such as malnutrition, diabetes, obesity, severe anaemia, bacterial vaginosis and group B streptococcus infections, as well as pre-labour rupture of membranes, and caesarean sections may enhance the risk [13]. Thus, while infections need to be treated, the emerging challenge of ABR as well as other potential risks related to exposure to antibiotics during pregnancy and lactation call for precaution on how and when antibiotics should be prescribed [14,15]. It is widely reported that intestinal microbiota are major determinants of health throughout infancy, childhood and later in adulthood [16]. The colonisation of the infant’s gut is critical to its normal adaptation to extra uterine life, and disruption of this process may lead to increased susceptibility to diseases later in life [17]. It has been shown that the use of antibiotic during pregnancy delays infant’s final gut colonisation contributing to increased susceptibility to infections and immune-mediated diseases [18,19]. This might result in frequent antibiotic consumption in childhood, higher number of allergic reactions episodes, increased healthcare costs and ultimately risk of ABR [19].

Although the use of antibiotics for uncomplicated vaginal delivery is generally not recommended by the World Health Organization (WHO) [20], high use of antibiotics in these situations have been reported in some countries [21]. A study from India showed that 87% of women who had uncomplicated vaginal delivery received antibiotics and 28% were discharged from the hospital with prescribed antibiotics, regardless of the mode of delivery [22]. Data from the Lao People’s Democratic Republic (Lao PDR or Laos) showed that among 300 women with uncomplicated vaginal delivery, 79% delivered at home, of those 25% used antibiotics, and that among women who delivered in hospitals, 79% received antibiotics [23]. 

Therefore, healthcare providers (HCPs) taking care of pregnant women, childbirth and children play a crucial role in avoiding unnecessary use of antibiotic. Every time doctors prescribe antibiotics to patients, they need to have a good knowledge regarding antibiotic to assure rational use and to avoid possible complications. Previous studies demonstrated that HCPs’ attitudes and knowledge determine the quality of prescription of antibiotics, and that understanding of HCP’s knowledge, attitudes and practices towards antibiotic use and ABR is a key to develop behaviour change interventions [24,25]. It has also been shown that ABR can be reduced by improving the knowledge, attitude, and practices of HCPs on antibiotic use and ABR [26]. Therefore, changing prescribing behaviour of HCPs is an essential component in the global campaign against ABR [27,28].

However, data on antibiotic use during pregnancy and childbirth is scarce in Laos and little is known about HCPs’ knowledge, attitudes, perceptions and practices regarding antibiotic use and ABR, but it is highly relevant to the nation as a response to the challenges described above. 

The aim of this study was to assess HCPs’ knowledge, attitudes, and practices and to explore their perceptions regarding antibiotic use and ABR in relation to pregnancy, childbirth, and children less than two years of age in two provinces of Laos. Our study, by providing essential baseline data, has implications for planning of future interventions aiming to improve antibiotic use in Laos, and in the long term to contain ABR. It is also relevant to many other settings with similar situations.

## 2. Results

### 2.1. Socio-Demographic Characteristics of Participants

In total, 217 HCPs participated in the quantitative part, including 107 participants in Feuang district (rural area) and 110 in Vangvieng district (urban area) in Vientiane province. There were no statistically significant differences between the two study sites regarding participants’ characteristics, except for median age and distribution of HCPs across practice types. Compared to Feuang district, HCPs in Vangvieng were significantly older and worked in lower proportion at health centres (26% vs. 55%) and in higher proportion (65% vs. 39%) at district hospitals (Table 1).

### 2.2. Knowledge of Participants Regarding Antibiotics and Antibiotic Resistance 

Most of the HCPs in both study districts could without any problems identify antibiotics from a presented matrix of various medicines. All participants (100%) correctly classified ampicillin, amoxicillin, and penicillin as antibiotics; however, only 68% recognised that cortisone is not an antibiotic. The majority of participants (97%) agreed that some antibiotics may have teratogenic effects and should be carefully considered before prescribing to pregnant women. The proportion of participants who replied that antibiotic use may cause side-effects and can contribute to ABR was significantly higher in Vangvieng than in Feuang district (100% vs. 94% and 94% vs. 85%).

The majority of participants (94%) had heard the term “antibiotic resistance”, knew that extensive use of antibiotics in society increases the risk of development and spread of resistance (91%), that the unnecessary use of antibiotics can make them ineffective in long term (89%), and that antibiotic course cannot be interrupted in the middle, even when symptoms of sick patients are improving (76%). Around one-third of participants (32%) replied that people cannot become resistant to antibiotics. The majority of participants (80%) agreed that today ABR is a big problem in their practice, 67% agreed that ABR is a problem in Laos and 58% that it is so worldwide (Table 2).

### 2.3. Attitudes of Participants towards Antibiotic Use and Resistance

The majority of participants (93%) agreed with the statement “patient non-adherence to the therapy is contributing to ABR”. Half of the HCPs thought that their prescribing practice is not contributing to ABR. There were no statistically significant differences between the two study districts regarding participants’ attitudes towards antibiotic use and ABR (Table 3).

### 2.4. Reported Practice of Antibiotics Prescribing/Dispensing 

Eighty-five percent (184/217) of participants replied that they prescribed/dispensed antibiotics to a patient during the previous week, and of those, 67% (n = 123) had prescribed/dispensed antibiotics more than four times. Antibiotics were prescribed/dispensed in the highest proportions for fever (69%), sore throat (63%) and cough (51%). Tonsillitis (44%), pneumonia (15%) and pharyngitis (10%) were the most common indications for which antibiotics were given. The most common antibiotic prescribed was amoxicillin, penicillin (phenoxymethyl penicillin), ampicillin, and erythromycin. The majority of patients (65%) received antibiotics for 5 days, 25% for 7 days and 8% for 3 days. Among other medicines, paracetamol was the most commonly prescribed/dispensed (92%).

### 2.5. Reported Practice of Antibiotics Prescribing/Dispensing during Pregnancy and Delivery

Around two-thirds (67%) of all participants replied that they would prescribe/dispense antibiotics for uncomplicated vaginal delivery without episiotomy. The proportion of HCPs prescribing/dispensing antibiotics for this indication was significantly higher in Feuang than in Vangvieng district (81% vs. 54%).

Sixty-five percent of participants reported that they followed the common practice in their health facility to prescribe/dispense antibiotics, and 23% explained that (even when it was not necessary) they prescribed because of the pressure from the patient. The proportion of HCPs prescribing/dispensing antibiotics due to concern about patients getting an infection was significantly higher in Feuang than in Vangvieng district (92% vs. 83%). Conversely, the proportion of antibiotics prescribed/dispensed due to pressure from patients was significantly higher in Vangvieng than in Feuang district (29% vs. 16%). Only a small fraction of all HCPs participated in antibiotic education activities and the proportion was significantly higher in Feuang than in Vangvieng district (13% vs. 5%) (Table 4).

### 2.6. Comparison of Participants’ Knowledge and Reported Practice Scores Regarding Antibiotic Use and Resistance in Both Study Districts

The total scores of participants’ knowledge and reported practice regarding antibiotic use and ABR were above average (total scores higher than the mean scores) in 64% and 41% of all participants, respectively. There were no statistically significant differences in the knowledge and practice scores regarding antibiotic use and ABR between the two study districts (Table 5).

### 2.7. Qualitative Findings

#### 2.7.1. Socio-Demographic Characteristics of Participants

In total, 30 HCPs and key stakeholders at different levels of health facilities in Vientiane province and Vientiane capital participated in the individual qualitative interviews, including nine participants at central level, nine at provincial level, and 12 at district level. The participants comprised nine specialists (six obstetricians-gynaecologists (OG) and three other specialists), six medical doctors (MD)/assistant doctors (AD), nine midwives (MW)/nurses (N), and six pharmacists (P)/assistant pharmacists (AP). The participants held various positions including directors of provincial hospital/provincial health department/district hospital/district health office, heads of health centres, obstetrics-gynaecology/antenatal care (ANC) team leaders and other technical staffs. The mean age of participants was 44; 67% were females; 93% were Lao ethnic, and 7% were Hmong. 

The findings from the individual interviews are presented under each main theme with quotes from the participants to illustrate the findings. Three main themes were identified as follows: (i) perceptions of antibiotics prescribing; (ii) experience with ABR and patient-doctor interaction and communication; and (iii) opinions about sources of information and educational intervention on antibiotic use in relation to pregnancy, childbirth and children under two.

#### 2.7.2. Perceptions of Antibiotics Prescribing 

##### Antibiotics Prescribed during Pregnancy and Delivery

The majority of participants stated a wider range of indications such as respiratory infection, urinary tract infection, or reproductive tract infection, as well as prophylactic provision in cases of caesarean section or uncomplicated vaginal delivery with episiotomy to prevent post-partum infection.

*“We prescribe antibiotics for all cases before performing caesarean section, delivery with episiotomy cases, perineal tear or premature rupture of membranes to prevent post-partum infection”*

(Female MD, 43 years old)

Some participants mentioned that the main reason for giving antibiotics to women with uncomplicated vaginal delivery was lack of confidence in proper sterilisation of delivery room and equipment. Moreover, HCPs doubted that patients understood well the provided instructions on how to manage wounds after the discharge.

*“The main reason that I prescribe antibiotics to pregnant women is to prevent and treat infection in uncomplicated vaginal delivery cases because we are afraid that the delivery room in the hospital is not guaranteed to be sterile”*

(Male OG, 55 years old)

*“In cases of uncomplicated vaginal delivery, during discharge from the hospital I prescribe amoxicillin tablets for 5 days to prevent post-partum infection as I am afraid that the patients did not understand properly about our health education provision regarding personal hygiene to keep their wounds clean”*

(Female MD, 43 years old)

Only few participants stated that they did not prescribe any antibiotic during uncomplicated vaginal delivery.

*“In cases of uncomplicated vaginal delivery, I do not prescribe any antibiotics, following the guidelines of the WHO that the use of antibiotics is not recommended for uncomplicated vaginal delivery”*

(Female MD, 53 years old)

##### Antibiotics Prescribed for Children under Two Years of Age

The majority of participants mentioned that they always prescribed/dispensed antibiotics for children with respiratory infections such as pharyngitis, tonsillitis, or pneumonia. Nearly half stated that they prescribed/dispensed antibiotics to children with skin infection, wounds, or diarrhoea.

*“For children under two, I dispense antibiotics in case of respiratory infection such as pharyngitis, tonsillitis, skin infection, leucocytosis, trauma or others”*

(Male P, 28 years old)

*“The majority of children received antibiotics because of diarrhoea, which is a seasonal disease that mostly occurred in summer”*

(Female N, 50 years old)

#### 2.7.3. Experience with Antibiotic Resistance and Patient–Doctor Interaction and Communication 

##### Experience with Antibiotic Resistance

Half of participants reported that they observed ABR in their daily practice and thought that it was a health problem; they were concerned about patients commonly self-medicating with antibiotics to treat e.g., fever or cough before seeing a doctor.

*“I think that antibiotic resistance is a health problem. Sometimes patients self-medicate with antibiotics when they get fever; if they could not recover then they come to see doctors. Since bacteria are resistant to antibiotics, doctors need to prescribe higher doses of antibiotics”*

(Male P, 29 years old)

Few participants mentioned that they were not sure if bacteria are resistant to antibiotics, as they did not perform bacterial culture to confirm AMR in their health facility.

*“It is not easy to identify if bacteria are resistant or not, because we did not do investigations of drug resistance. However, there was a report about bacteria that caused Typhoid fever were resistant to antibiotics. For other bacteria I had never heard”*

(Female MD, 50 years old)

##### Patient–Doctor Interaction and Communication

Almost all participants declared that they provide information on antibiotic properties, use, possible adverse events and other relevant issues. However, a few participants said that they do not inform patients about bacterial resistance, as they themselves need to gain more knowledge.

*“If the result of blood test (such as leucocyte count) show bacterial infection we will explain to patients about which kind of antibiotics to be given, what are their properties, how much doses, why we need to use this drug. So we need antibiotic knowledge to explain to patients about the effectiveness of antibiotics, their mechanism of action, how to make patients feel safe on the treatment”*

(Female OG, 50 years old)

Half of participants perceived that patients had some knowledge about antibiotics from social media or drug advertising. Others thought that patients did not have any knowledge about antibiotics and proposed that a majority of patients believed that antibiotics could treat a range of infections including a common flu or cough. They also cautioned that some patients may not complete the course of antibiotic. Few participants thought that antibiotic knowledge of patients depend on the level of education.

*“The majority of patients including pregnant women asked to buy antibiotics when they got flu or cough, as they thought that antibiotics can treat every illness. I thought that patients didn’t know how to use antibiotics for instance when they got sick and went to see doctors, who prescribed antibiotics to them for 5–7 days, but they took only 2–3 days then stopped using the medicines. We need to explain to patients that inappropriate use of antibiotic may cause antibiotic resistance”*

(Male AP, 44 years old)

*“I think that antibiotic use depends on patient’s knowledge. Some patients knew well how to use antibiotic and followed the doctor’s recommendation, but some had difficulty to understand as we have to explain to them many times how to take antibiotic, especially for minority ethnic group”*

(Male P, 57 years old)

#### 2.7.4. Opinions about Sources of Information and Educational Intervention on Antibiotic Use in Relation to Pregnancy, Childbirth and Children under Two

##### Sources of Information on Antibiotic Treatment

Several participants stated that their source of information about antibiotics were national treatment guidelines, the WHO guidelines, lectures or training, magazines, scientific journals, or internet.

*“The majority of information on antibiotics that I got was based on national treatment guidelines, and the guidelines from WHO”*

(Female OG, 37 years old)

*“I received health information on antibiotic use from doctors who gave lectures based on the guidelines of paediatrics and obstetrics-gynaecology associations”*

(Male MD, 55 years old)

The majority of participants stated that guidelines on antibiotic use during pregnancy, delivery and for children under two would be valuable for their daily practice and would allow them to provide correct treatment and sufficient health information to patients.

*“It would be good if antibiotic guideline is available, as we can follow the guideline how to treat, no need to ask for help from others, no need to try to get other sources of information on antibiotic use”*

(Female AP, 43 years old)

*“If antibiotic guideline is available it would be able to give correct information to pregnant women to understand better”*

(Female OG, 37 years old)

Most of the participants mentioned that they did not receive any specific training about antibiotic treatment in relation to pregnancy, childbirth and children under two. Only a few participants had attended training on rational medicine use and other topics.

*“It would be great if continuing professional development is available. In the past, there was no training program regarding antibiotic use. The training on mother and child health education did not focus on antibiotic use, but rather focus on the health care during pregnancy, delivery and postnatal care for mother and child”*

(Female MD, 53 years old)

##### Acceptability and Appropriateness of Educational Intervention 

Most of participants stated that they would like to attend training on antibiotic use in relation to pregnancy, childbirth and children under two, as they believed that it is the best way to improve their knowledge.

*“To improve HCPs’ knowledge, we need to attend training with trainers from the Ministry of Health to upgrade our knowledge on antibiotic use and other topics as well”*

(Male MD, 32 years old)

About half of participants said that they would like to improve patients’ knowledge by providing health information through training, advertisements, posters and brochures.

*“It would be better to educate general population or pregnant women regarding antibiotic use in relation to pregnancy, delivery and children under two by using advertising, posters, brochures, then training HCPs”*

(Male OG, 52 years old)

One participant said that training on antibiotic guidelines should be included in the medical university course. Another participant mentioned that advertising via social network is an option for educational intervention.

*“I think that we have to add antibiotic training into the learning program in the medical university to have more knowledge about antibiotic use before graduating”*

(Male AP, 44 years old)

*“I request to have posters, magazine, broadcast via radio about antibiotic use in pregnancy, childbirth and children under two or via social media like Facebook”*

(Female MW, 40 years old)

## 3. Discussion

To the best of our knowledge, this is the first study conducted in Laos that aims to assess knowledge, attitudes, practices of HCPs and to explore their perceptions regarding antibiotic use and ABR in relation to pregnancy, childbirth, and children under two. We found that one-third of HCPs had low knowledge about antibiotic use and ABR, two-thirds reported non-indicated antibiotics prescribing for uncomplicated vaginal delivery, and half of HCPs did not believe that their prescribing practice contributed to ABR. Importantly, the majority of HCPs had never participated in any antibiotic education activities. 

We observed significant differences in non-indicated prescription between the study areas with a higher proportion of antibiotic prescribed for uncomplicated vaginal delivery in Feuang than in Vangvieng district (81% vs. 54%). The reason was that the majority of HCPs, especially in the rural area, was concerned about patients developing infection-related complications. This was evident from our qualitative findings, where HCPs explained that the main reason for prescribing antibiotics in case of uncomplicated vaginal delivery was to prevent post-partum infection linked to the fear that the sterilisation of delivery room and equipment was not guaranteed and that the health education provided would not be well understood by patients. The results are in line with a previous study conducted in Laos in 2008 presenting that 79% of women who had uncomplicated vaginal delivery received antibiotics in hospitals [23]. Studies from other countries also reported that up to 60% of women with uncomplicated vaginal delivery received intrapartum antibiotics and were prescribed antibiotics at discharge [22,29]. According to the WHO, routine antibiotic prophylaxis is not recommended for women with uncomplicated vaginal delivery. The high rate of routine use of antibiotics for this indication without any specific risk factors has serious public health implications and contributes to development and spread of ABR [20]. The use of any antibiotic during pregnancy or delivery should be based on whether benefits outweigh the risk; every time HCPs prescribe, they should consider the potential benefit associated with antibiotic treatment and potential adverse effects associated with each medicine, and the development of future ABR which is a major concern in the world, as well as in Laos. 

The findings of our study revealed that 36% of HCPs had below average knowledge regarding antibiotic use and ABR and the level was comparable between the rural and urban area. This might be due to participants’ similar demographic characteristics including types of HCPs in both study districts. In addition, they underwent the same education and, as presented in the qualitative findings, received information on antibiotics from the national treatment guidelines, the WHO guidelines, lectures or training, magazines or scientific journals and from the internet. Although the majority of all participants reported that antibiotics are effective against bacterial infections, about one-third thought that common cold is normally caused by bacteria; this may contribute to HCPs’ decision to prescribe antibiotics to patients with a common cold. Previous studies from other countries also showed that about 20–60% of the participants believed that antibiotics should be prescribed for cold of viral aetiology [26,30,31]. Knowledge of physicians is an important factor that affects prescribing of antibiotics [10,31,32]. However, even if knowledge is good, this does not always translate into good prescribing practices [33]. There is an immediate need to change the way of prescribing and using antibiotics, especially for pregnant women and children.

An interesting aspect was that half of our participants did not think that their own prescribing affected ABR; this could result in prescribing behaviours that actually promoted ABR. It has been shown that the majority of participants declared that the previous use of antibiotics increased the person’s risk of acquiring drug-resistant infection [34]. In addition, when HCPs other than MDs, prescribed antibiotics they recommended broad spectrum antibiotics and shorter treatment time to a higher extent; this, unfortunately, drive ABR [35]. Regarding the awareness about the current scope of ABR, our study result showed that many participants (80%) thought that ABR is a big problem in their workplace, more than in the country (67%) and in the world (58%). In addition, in the qualitative findings, participants mentioned that they observed ABR in their daily practice and thought that it was a health problem. Some participants, however, were not sure if bacteria are resistant to antibiotics or not, as they did not perform bacterial culture to confirm ABR in their health facility. A previous study in Laos showed that two-thirds of the participants thought that ABR is a big problem in their workplace, less than in the country (75%) and in the world (84%) [36]. Studies from other countries also showed that the majority of participants perceived ABR as an important problem, but to a lesser extent in their own practice than at the national or global level [10,24,32,37]. It might be that the results from different settings/countries may differ due to the HCPs’ level of education and the standard of care in the different tiered hospitals or practices. The findings could have a potential impact on patient care and infection control activities (such as hand-washing/disinfection, and cleaning/sterilisation of equipment), as HCPs are more likely to pay attention to the ABR while selecting antibiotics if they consider ABR as a problem in their workplace. In addition, there is a need for the use of laboratory services and support from infectious disease experts to guide antibiotic therapy in order to improve the awareness on local ABR [37].

Our findings showed that nearly two-thirds of all participants reported that they prescribed antibiotics by following the common practice in their health facility. One-quarter prescribed antibiotics due to the perceived pressure from the patient, although the HCPs did not think it was necessary. Additionally, our qualitative findings showed that half of the HCP participants mentioned that the majority of their patients believed that antibiotics can treat every illness and want to use antibiotics even in cases of flu or cough. Studies from other countries also reported that most physicians identified that they felt being under pressure if the patients expected an antibiotic prescription, even for treating viral infections; this may contribute to antibiotic overuse and ABR [9,32,34]. It has been reported that some doctors may feel pressured to prescribe antibiotics to ensure patient satisfaction, or some physicians might be afraid of losing their patients if they want to use antibiotics [38,39]. However, an antibiotic prescription may not necessarily ensure patient satisfaction; studies have shown that proper communication is considered more important by patients [32,38,39]. The high expectation about antibiotics from patients might be a consequence of their minimal understanding of antibiotics and antibiotic side effects. Thus, it is necessary to educate the general public, particularly pregnant women and mothers of young children through community-targeted media information, and the ANC visits may be a good opportunity to implement the educational programs on antibiotic use among pregnant women [32,40]. In addition, communication training of HCPs is needed to facilitate decision making and empower doctors to decline antibiotic requests [41]. 

Only 9% of HCPs reported that they had participated in antibiotic education. This was confirmed during the qualitative interviews, when most of the participants mentioned that they did not receive any specific training about antibiotic treatment and that the continuous professional development was not in place. Most of HCPs requested to have more educational programs regarding antibiotic prescribing, especially in relation to pregnancy, childbirth, and young children, as they believed that it is the best way to improve their knowledge. Studies from other countries also showed that the majority of participants needed treatment guidelines and educational programs on antibiotics prescribing [32,42]. A study among doctors in Laos showed that they lacked access to information about prescribing antibiotics and 60% claimed that they had not enough information about antibiotics [36]. Therefore, policymakers should consider developing antibiotic prescribing guidelines focusing on infections management in pregnancy, childbirth, and for young children. In addition, the guidelines should emphasise the damage that can be caused by over antibiotic prescribing and the ABR crisis. We believe that implementation of these tools could improve the attitudes, perceptions and behaviours of prescribers. 

The strengths of this study were that the quantitative data were triangulated with qualitative findings. The study has provided useful information on HCPs’ perceptions, knowledge, attitudes, and reported practices regarding antibiotic use and ABR. The quantitative and qualitative findings gained from this study can be used to develop educational interventions to promote appropriate antibiotic use. However, every study needs to be seen in the context of its limitations. First, the quantitative part was conducted in only two districts of Vientiane province, and although they represented different ethnic minority population and different settings (rural and urban), may not be representative of all the country. Second, all provided responses were self-reported and might not reflect the actual practice, since it was not possible to ascertain whether what the HCPs reported was an accurate description of their real practice of antibiotic use, thus, the possibility of reporting bias cannot be excluded. Third, all responses were based on memory of past events that may affect accurate recall, resulting in both under-and over-reporting.

## 4. Materials and Methods

### 4.1. Study Design

This is a mixed method, cross-sectional study containing quantitative and qualitative components. The presented study is a part of the CAREChild (Containment of Antibiotic REsistance—measures to improve antibiotic use in pregnancy, childbirth, and young children) project conducted in Laos [43]. 

### 4.2. Study Settings

The study was carried out in Laos, a lower middle-income country in Southeast Asia with approximately 7 million inhabitants and relatively low population density [44]. Laos has 17 provinces, one capital city-Vientiane, 148 districts and 8459 villages. Vientiane capital, located in the middle part of Laos, has 9 districts with the total population of 820,000 inhabitants [45]. Lao population is relatively young with about 32% being under the age of 15 years, 64% aged 15–64 and 4% being 65 and older [45]. Around two-thirds of the population lives in rural areas and relies on agricultural work, specifically rice farming. 

The quantitative part of the study took place in Vientiane province in the middle part of Laos. Vientiane province has 11 districts with the total population of 450.000 inhabitants. Two districts (Feuang and Vangvieng) were purposively selected as study areas based on variation in participants background characteristics, representing different ethnic minority populations, and also for feasibility reasons. Feuang district is a rural area, and its health care system is composed of 1 district hospital, 6 health centres, 8 private pharmacies and 107 HCPs. Vangvieng district is an urban area and has 1 district hospital, 5 health centres, 10 private pharmacies and 110 HCPs [46]. 

The qualitative part using individual interviews was carried out in Thoulakhom district in Vientiane province and Maternal Child-Newborn Hospital (MCNH) in Vientiane capital, which were outside of the study areas. Thoulakhom district was purposively selected for feasibility reasons; it has 1 district hospital and 5 health centres. The MCNH is 1 of the 5 central hospitals, and is a university hospital, where women from different parts of the country come for ANC and consultation. 

### 4.3. Study Population 

All HCPs (medical doctors/assistant doctors, midwives/nurses, and pharmacists/ assistant pharmacists) who prescribe or dispense antibiotics at the health facilities (district hospitals and health centres) and private pharmacies in both study sites were invited to participate in the quantitative study. They were identified from the health staff registry available at the district health offices. 

Representatives of HCPs and policy makers in Vientiane province (Vientiane provincial health department/hospital, Thulakhom district hospital with 2 health centres), and representatives from central level such as Ministry of Health, University of Health Sciences, obstetrics-gynaecology association in addition to the MCNH in Vientiane capital were invited to take part in the individual interviews. They were purposively selected upon the identification in the local health staff name list.

### 4.4. Development of Data Collection Tools

Structured questionnaires were developed based on previously used tools within and outside the project group and adapted for the different target groups to collect data on knowledge, attitudes and reported practices regarding antibiotic use and ABR in relation to pregnancy, delivery and early childhood [43]. The study tools were pre-tested in Phonehong district (Vientiane province) and Sangthong district (Vientiane capital) with 30 HCPs working across 2 district hospitals, 3 health centres, and 3 private pharmacies to check for clarity and comprehension of the questions. Based on the pre-test results, the questionnaires were adjusted and finalised and the refresh training was provided to all data collectors. 

A qualitative interview guide was developed and pre-tested in Phonehong district with 4 HCPs working across 1 district hospital, 1 health centre, and 1 private pharmacy to ensure that the questions were understood. This tool was used to explore HCPs’ perceptions towards antibiotics prescribing, ABR, patient-doctor interaction and communication, sources of information and educational intervention on antibiotic use in relation to pregnancy, childbirth and children under two. Based on the feedback from the pre-test the interview guide was adjusted and finalised. 

All data collection tools were developed in English, translated to Lao and pre-tested. Necessary adjustments were performed in Lao language. The final versions of the tools were then back-translated to English to check for consistency in the translation.

### 4.5. Data Collection 

Quantitative data was collected from September to November 2019. All included HCPs were informed about the purpose of the study and consented to participate. The interview was performed at a convenient place such as a meeting room or their office. Digital capture was used to collect data on social demographic characteristics, knowledge, attitudes, and reported practices of HCPs regarding antibiotic use and ABR. 

The individual qualitative interviews were carried out in the same time period by 1 interviewer and 1 note taker/audio-recorder, who were research team members. The participants were interviewed at the time and location of their choice, mostly at their office. Each interview lasted approximately 40 min. Sessions were continued until data saturation was reached (limited or no new information was obtained) [47].

### 4.6. Data Management and Analysis

Data were managed by using Research Electronic Data Capture (REDCap) tool hosted at Karolinska Institutet [48]. Data were collected via tablets and checked for completeness daily. The data was analysed using STATA version 14 (Stata Corporation; College Station, TX, USA). Categorical variables were presented as frequencies, percentages and proportions, while continuous variables were presented in median, means and standard deviations. Chi-square test (χ^2^-test) was used to compare two categorical variables, and Mann–Whitney was used to compare continuous variables, particularly comparing differences between Feuang (rural area) and Vangvieng district (urban area); a *p* value < 0.05 was considered statistically significant. 

The reported practice and knowledge scores were calculated based on correct (1 point) or incorrect (0 point) answer received from HCPs regarding their practice and knowledge. The points were summarised, and the mean score was calculated. If the total score was greater than the mean the reported practice and knowledge were classified as “above average” and if less or equal to mean they were classified as “below average”. 

The attitudes of HCPs towards antibiotic use and ABR were assessed by using a 5-point Likert like scale based on the statements whose responses included “strongly agree”, “agree”, “don’t know”, “disagree” and “strongly disagree”. We combined “strongly agree” and “agree” answers to define “agree”. We also performed the same combination for “strongly disagree” and “disagree” to define “disagree”.

The audio-records and notes from the individual qualitative interviews were transcribed verbatim. The Lao transcripts were entered into the computer (as a word file) and analysed using qualitative content analysis [49]. The validity of the analysis was based on the triangulation of information, and the independent coding was performed by 3 researchers, who are among the authors, to identify the similarities and differences of perceptions.

## 5. Conclusions

This study indicated that one-third of HCPs had low ABR-related knowledge, two-thirds reported prescribing antibiotics for uncomplicated vaginal delivery, and half claimed their prescribing of antibiotics did not contribute to ABR. Unnecessary, non-indicated antibiotics prescribing coupled with low knowledge among a substantial number of HCPs may be an underlying reason for the development and spread of ABR. Therefore, continuous antibiotic education for HCPs and regular supervision are recommended in order to improve the use of antibiotics in relation to pregnancy, childbirth, and young children in health facilities.

## Figures and Tables

**Table 1:** Socio-demographic characteristics of participants in the two study districts.

Socio-Demographic Characteristics	Total (n = 217)	Feuang (n = 107)	Vangvieng (n = 110)	*p* Value
	n (%)	n (%)	n (%)	
Age median (IQR)	35 (29–49)	32 (27–45)	39 (31–50)	0.001 **
Gender				n/s
Male	67 (31)	38 (36)	29 (26)	
Female	150 (69)	69 (64)	81 (74)	
HCP types				n/s
Medical doctor/Assistant doctor	46 (21)	21 (19)	25 (23)	
Midwife/Nurse	110 (51)	54 (48)	56 (51)	
Pharmacist/Assistant pharmacist	34 (16)	18 (17)	16 (14)	
Drug seller	11 (5)	5 (5)	6 (5)	
Other #	16 (7)	9 (8)	7 (6)	
Practice types				<0.001 *
District hospital	113 (52)	42 (39)	71 (65)	
Health centre	88 (41)	59 (55)	29 (26)	
Private pharmacy	16 (7)	6 (6)	10 (9)	

Note: * significant differences for the χ^2^-test and ** Mann–Whitney test; n/s, non-significant differences. Abbreviations: IQR, interquartile range; # Other, dentist, laboratory technician.

**Table 2:** Knowledge of participants regarding antibiotic effectiveness and resistance.

AB Effectiveness and Resistance Variables	Total (n = 217)	Feuang (n = 107)	Vangvieng (n = 110)	*p* Value
	n (%)	n (%)	n (%)	
AB are effective against bacterial infections (correct)	185 (85)	91 (85)	94 (85)	n/s
AB are effective against viral infections (incorrect)	64 (29)	28 (26)	36 (33)	n/s
Common cold is normally caused by bacteria (incorrect)	65 (30)	30 (28)	35 (32)	n/s
AB use may cause side effects (correct)	211 (97)	101 (94)	110 (100)	0.013 *
Some AB may have teratogenic effect and their use for pregnant women should be carefully considered (correct)	211 (97)	103 (96)	108 (98)	n/s
AB use can contribute to ABR (correct)	194 (89)	91 (85)	103 (94)	0.040 *
Bacteria can become resistant to AB (correct)	183 (84)	85 (79)	98 (89)	n/s
ABR can spread from animals to humans (correct)	79 (36)	42 (39)	37 (34)	n/s
Today, ABR is a big problem in your practice #	173 (80)	83 (78)	90 (82)	n/s
Today, ABR is a big problem in Laos #	146 (67)	73 (68)	73 (66)	n/s
Today, ABR is a big problem in the world #	126 (58)	64 (60)	62 (56)	n/s

Note: * significant differences for the χ^2^-test; n/s, non-significant differences; **#** neither correct nor incorrect answer. Abbreviations: AB, antibiotic; ABR, antibiotic resistance.

**Table 3:** Attitudes of participants towards antibiotic use and resistance.

Statements	Total (n = 217)	Feuang (n = 107)	Vangvieng (n = 110)
	n (%)	n (%)	n (%)
I would prescribe/dispense an AB for an adult patient with runny nose, cough or fever to help him/her to get better quickly			
Agree	84 (39)	39 (36)	45 (41)
Disagree	131 (60)	68 (64)	63 (57)
Don’t know	2 (1)	0 (0)	2 (2)
I would normally give AB to a child with a common cold			
Agree	59 (27)	23 (22)	36 (33)
Disagree	156 (72)	83 (77)	73 (66)
Don’t know	2 (1)	1 (1)	1 (1)
I prefer to store AB at home, just in case I need them			
Agree	63 (29)	32 (30)	31 (28)
Disagree	154 (71)	75 (70)	79 (72)
Don’t know	0 (0)	0 (0)	0 (0)
I would use leftover AB for any of my family member			
Agree	28 (13)	9 (8)	19 (17)
Disagree	189 (87)	98 (92)	91 (83)
Don’t know	0 (0)	0 (0)	0 (0)
My prescribing practice is not contributing to ABR			
Agree	109 (50)	55 (52)	54 (49)
Disagree	91 (42)	41 (38)	50 (46)
Don’t know	17 (8)	11 (10)	6 (5)
Patient non-adherence to the therapy is contributing to ABR			
Agree	201 (93)	99 (92)	102 (93)
Disagree	14 (6)	6 (6)	8 (7)
Don’t know	2 (1)	2 (2)	0 (0)

Note: agree, strongly agree/agree; disagree, strongly disagree/disagree. Abbreviations: AB, antibiotic; ABR, antibiotic resistance.

**Table 4:** Reported practices of participants regarding antibiotic prescribing/dispensing.

Variables	Total (n = 217)	Feuang (n= 107)	Vangvieng (n = 110)	*p* Value
	n (%)	n (%)	n (%)	
*Mode of delivery*				
Uncomplicated vaginal delivery without episiotomy	146 (67)	87 (81)	59 (54)	0.001 *
Uncomplicated vaginal delivery with episiotomy	206 (95)	103 (96)	103 (94)	n/s
Caesarean section	210 (97)	104 (97)	106 (96)	n/s
*Reasons for AB use*				
Prescribed AB due to pressure from patient	49 (23)	17 (16)	32 (29)	0.003 *
Follow common practice	140 (65)	65 (61)	75 (68)	n/s
Concern for infections	190 (88)	99 (93)	91 (83)	0.029 *
*AB education/information/spectrum AB*				
Participated in AB education	19 (9)	14 (13)	5 (5)	0.023 *
Provided AB information for patients	196 (90)	101 (94)	95 (86)	n/s
Prefer broad spectrum over narrow spectrum AB	61 (28)	27 (25)	34 (31)	n/s

Note: * significant differences for the χ^2^-test; n/s, non-significant differences. Abbreviations: AB, antibiotic.

**Table 5:** Participants’ knowledge and reported practice scores regarding antibiotic use and resistance in both study districts.

Scores	Total (n = 217)	Feuang (n = 107)	Vangvieng (n = 110)	*p* Value
	n (%)	n (%)	n (%)	
Knowledge				n/s
Below average (<=mean)	79 (36)	42 (39)	37 (33.6)	
Above average (>mean)	138 (64)	65 (61)	73 (66.4)	
Mean (SD)	18 (2)	17 (2)	18 (2)	
Min:Max	10:22	10:22	14:22	
Reported practice				n/s
Below average (<=mean)	129 (59)	59 (55)	70 (64)	
Above average (>mean)	88 (41)	48 (45)	40 (36)	
Mean (SD)	10 (1)	10 (1)	10 (1)	
Min:Max	5:13	6:13	5:12	

Note: Abbreviations: SD, standard deviation; n/s, non-significant differences.

## Data Availability

Relevant data for this study are presented in the tables. Any further data are available upon request from the corresponding author.

## References

[1] World Health Organization (2015). Global Action Plan on Antimicrobial Resistance.

[2] O’Neill J. (2014). Antimicrobial Resistance: Tackling a Crisis for the Health and Wealth of Nations.

[3] World Health Organization (2018). Antibiotic-Resistance [Internet]. http://www.who.int/en/news-room/fact-sheets/detail/antibiotic-resistance.

[4] World Health Organization (2011). The World Medicines Situation.

[5] Rodrigues A.T., Roque F., Falcão A., Figueiras A., Herdeiro M.T. (2013). Understanding physician antibiotic prescribing behaviour: A systematic review of qualitative studies. Int. J. Antimicrob. Agents..

[6] Marzan M., Islam D.Z., Lugova H., Krishnapillai A., Haque M., Islam S. (2021). Knowledge, attitudes, and practices of antimicrobial uses and resistance among public university students in Bangladesh. Infect. Drug Resist..

[7] Muhie O.A. (2019). Antibiotic use and resistance pattern in Ethiopia: Systematic review and meta-analysis. Int. J. Microbiol..

[8] Landstedt K., Sharma A., Johansson F., Lundborg C.S., Sharma M. (2017). Antibiotic prescriptions for inpatients having non-bacterial diagnosis at medicine departments of two private sector hospitals in Madhya Pradesh, India: A cross-sectional study. BMJ Open..

[9] Nair M., Tripathi S., Mazumdar S., Mahajan R., Harshana A., Pereira A., Jimenez C., Halder D., Burza S. (2019). Knowledge, attitudes, and practices related to antibiotic use in Paschim Bardhaman district: A survey of healthcare providers in West Bengal, India. PLoS ONE.

[10] Thriemer K., Katuala Y., Batoko B., Alworonga J.P., Devlieger H., Van Geet C., Ngbonda D., Jacobs J. (2013). Antibiotic prescribing in DR Congo: A knowledge, attitude and practice survey among medical doctors and students. PLoS ONE.

[11] Keohavong B., Vonglokham M., Phoummalaysith B., Louangpradith V., Inthaphatha S., Kariya T., Saw Y.M., Yamamoto E., Hamajima N. (2019). Antibiotic prescription for under-fives with common cold or upper respiratory tract infection in Savannakhet Province, Lao PDR. Trop. Med. Health.

[12] Sekikubo M., Hedman K., Mirembe F., Brauner A. (2017). Antibiotic over consumption in pregnant women with urinary tract symptoms in Uganda. Clin. Infect. Dis..

[13] World Health Organization (2021). WHO Recommendation on Routine Antibiotic Prophylaxis for Women Undergoing Operative Vaginal Birth.

[14] Martinez de Tejada B. (2014). Antibiotic use and misuse during pregnancy and delivery: Benefits and risks. Int. J. Environ. Res. Public Health.

[15] Mylonas I. (2011). Antibiotic chemotherapy during pregnancy and lactation period: Aspects for consideration. Arch. Gynecol. Obstet..

[16] Mackie R.I., Sghir A., Gaskins H.R. (1999). Developmental microbial ecology of the neonatal gastrointestinal tract. Am. J. Clin. Nutr..

[17] Walker W.A. (2017). The importance of appropriate initial bacterial colonization of the intestine in newborn, child, and adult health. Pediatr. Res..

[18] Smaill F.M., Grivell R.M. (2014). Antibiotic prophylaxis versus no prophylaxis for preventing infection after cesarean section. Cochrane Database Syst. Rev..

[19] American College of Obstetricians and Gynecologists (2011). ACOG practice bulletin No.120: Use of prophylactic antibiotics in labor and delivery. Obstet. Gynecol..

[20] World Health Organization (2015). World Health Organization Recommendations for Prevention and Treatment of Maternal Peripartum Infections.

[21] Bonet M., Ota E., Chibueze C.E., Oladapo O.T. (2017). Routine antibiotic prophylaxis after normal vaginal birth for reducing maternal infectious morbidity. Cochrane Database Syst. Rev..

[22] Sharma M., Sanneving L., Mahadik K., Santacatterina M., Dhaneria S., Stålsby Lundborg C. (2013). Antibiotic prescribing in women during and after delivery in a non-teaching, tertiary care hospital in Ujjain, India: A prospective cross-sectional study. J. Pharm. Policy Pract..

[23] Keohavong B., Sihavong A., Soukhaseum T., Oudomsak P., Souliyavong K., Soundavong K., Voradeth S., Kounnavong S., Houamboun K., Akkhavong K. Use of Antibiotics among Mothers after Normal Delivery in Two Provinces in Lao PDR. Proceedings of the Third International Conference on Improving Use of Medicines.

[24] Gonzalez-Gonzalez C., López-Vázquez P., Vázquez-Lago J.M., Piñeiro-Lamas M., Herdeiro M.T., Arzamendi P.C., Figueiras A., GREPHEPI Group (2015). Effect of physicians attitudes and knowledge on the quality of antibiotic prescription: A Cohort study. PLoS ONE.

[25] Labi A.K., Obeng-Nkrumah N., Bjerrum S., Aryee N.A.A., Ofori-Adjei Y.A., Yawson A.E., Newman M.J. (2018). Physicians’ knowledge, attitudes, and perceptions concerning antibiotic resistance: A survey in a Ghanaian tertiary care hospital. BMC Health Serv. Res..

[26] Zulu A., Matafwali S.K., Banda M., Mudend S. (2020). Assessment of knowledge, attitude and practices on antibiotic resistance among undergraduate medical students in the school of medicine at the University of Zambia. Int. J. Basic Clin. Pharmacol..

[27] Lopez-Vazquez P., Vazquez-Lago J.M., Figueiras A. (2012). Misprescription of antibiotics in primary care: A critical systematic review of its determinants. J. Eval. Clin. Pract..

[28] Liu C., Liu C., Wang D., Zhang X. (2019). Knowledge, attitudes and intentions to prescribe antibiotics: A structural equation modeling study of primary care institutions in Hubei, China. Int. J. Environ. Res. Public Health.

[29] Stokholm J., Schjørring S., Pedersen L., Bischoff A.L., Følsgaard N., Carson C.G., Chawes B.L.K., Bønnelykke K., Mølgaard A., Krogfelt K.A. (2013). Prevalence and predictors of antibiotic administration during pregnancy and birth. PLoS ONE.

[30] Azevedo M.M., Pinheiro C., Yaphe J., Baltazar F. (2009). Portuguese students’ knowledge of antibiotics: A cross-sectional study of secondary school and university students in Braga. BMC Public Health.

[31] Khan A.K., Banu G., Reshma K. (2013). Antibiotic resistance and usage—A survey on the knowledge, attitude, perceptions, and practices among the medical students of a Southern Indian teaching hospital. J. Clin. Diagn. Res..

[32] García C., Llamocca L.P., García K., Jiméne A., Samalvides F., Gotuzzo E., Jacobs J. (2011). Knowledge, attitudes and practice survey about antimicrobial resistance and prescribing among physicians in a hospital setting in Lima, Peru. BMC Clin. Pharmacol..

[33] Aragoneses J., Suárez A., Rodríguez C., Algar J., Aragoneses J.M. (2021). Knowledge, attitudes, and practices among dental practitioners regarding antibiotic prescriptions for pregnant and breastfeeding women in the Dominican Republic. Antibiotics.

[34] Al-Homaidan H.T., Barrimah I.E. (2018). Physicians’ knowledge, expectations, and practice regarding antibiotic use in primary health care. Int. J. Health Sci..

[35] Auta A., Hadi M.A., Oga E., Adewuyi E.O., Abdu-Aguye S.N., Adeloye D., Strickland-Hodge B., Morgan D.J. (2019). Global access to antibiotics without prescription in community pharmacies: A systematic review and meta-analysis. J. Infect..

[36] Quet F., Vlieghe E., Leyer C., Buisson Y., Newton P.N., Naphayvong P., Keoluangkhot V., Chomarat M., Longuet C., Steenkeste N. (2015). Antibiotic prescription behaviours in Lao People’ s Democratic Republic: A knowledge, attitude and practice survey. Bull. World Health Organ..

[37] Chaw P.S., Höpner J., Mikolajczyk R. (2018). The knowledge, attitude and practice of health practitioners towards antibiotic prescribing and resistance in developing countries—A systematic review. J. Clin. Pharm..

[38] Farley E., Stewart A., Davies M.A., Govind M., van den Bergh D., Boyles T.H. (2018). Antibiotic use and resistance: Knowledge, attitudes and perceptions among primary care prescribers in South Africa. S. Afr. Med. J..

[39] Johana E., Prawitasari H., Sri S., Sulanto S., Danu S., Budiono S. (1996). Interactional group discussion: Results of a controlled trial using a behavioral intervention to reduce the use of injections in public health facilities. Soc. Sci. Med..

[40] Bulabula A.N.H., Dramowski A., Mehtar S. (2020). Antibiotic use in pregnancy: Knowledge, attitudes and practices among pregnant women in Cape Town, South Africa. J. Antimicrob. Chemother..

[41] Saliba-Gustafsson E.A., Hampton A.D., Zarb P., Orsini N., Michael A., Borg M.A., Stalsby Lundborg C. (2019). Factors associated with antibiotic prescribing in patients with acute respiratory tract complaints in Malta: A 1-year repeated cross-sectional surveillance study. BMJ Open..

[42] Om C., Vlieghe E., Mclaughlin J.C. (2016). Antibiotic prescribing practices: A national survey of Cambodian physicians. Am. J. Infect. Control..

[43] Machowska A., Sihavong A., Eriksen J., Vongsouvath M., Marrone G., Sychareun V., Hanson C., Keohavong B., Brauner A., Mayxay M. (2020). Containment of antibiotic REsistance--measures to improve antibiotic use in pregnancy, childbirth and young children (CAREChild): A protocol of a prospective, quasi experimental interventional study in Lao PDR. BMJ Open..

[44] World Bank The World Bank Data. https://data.worldbank.org/country/lao-pdr.

[45] Population and Housing Census 2015 (2015). Provisional Report.

[46] Vientiane Capital (2019). District Population Projections.

[47] Krueger R.A. (1998). Focus Groups: A Practical Guide for Applied Research.

[48] REDCap Research Electronic Data Capture. https://projectredcap.org/software/.

[49] Graneheim U.H., Lundman B. (2004). Qualitative content analysis in nursing research: Concepts, procedures and measures to achieve trustworthiness. Nurse Educ. Today.

